# Extracellular MicroRNAs as Intercellular Mediators and Noninvasive Biomarkers of Cancer

**DOI:** 10.3390/cancers12113455

**Published:** 2020-11-20

**Authors:** Blanca Ortiz-Quintero

**Affiliations:** Research Unit, Instituto Nacional de Enfermedades Respiratorias Ismael Cosío Villegas, Calzada de Tlalpan 4502, Colonia Sección XVI, Mexico City 14080, Mexico; boq@iner.gob.mx; Tel.: +52-55-54871705

**Keywords:** extracellular miRNAs, intercellular mediators, tumor development, tumor progression, non-invasive cancer biomarkers

## Abstract

**Simple Summary:**

There are an extensive number of publications regarding the role of endogenous miRNAs as regulators of gene expression in cancer. However, extracellular miRNAs have emerged as a novel mechanism of cell-to-cell communication in normal conditions and disease and have drawn a large amount of interest as regulators of gene expression and as potential non-invasive biomarkers in cancer. Despite this high interest and the abundance of research on the biology and role of extracellular miRNAs in cancer, they are not yet completely understood. The aim of this review is to highlight the relevant biological characteristics of extracellular miRNAs that enable them to function as intercellular mediators of gene expression regulation and provide the recently published evidence of the specific role of extracellular miRNAs in tumor development and progression.

**Abstract:**

MicroRNAs (miRNAs) are released by different types of cells through highly regulated mechanisms under normal and pathological conditions. These extracellular miRNAs can be delivered into recipient cells for functional purposes, acting as cell-to-cell signaling mediators. It has been discovered that cancer cells release miRNAs into their surroundings, targeting normal cells or other cancer cells, presumably to promote tumor development and progression. These extracellular miRNAs are associated with oncogenic mechanisms and, because they can be quantified in blood and other bodily fluids, may be suitable noninvasive biomarkers for cancer detection. This review summarizes recent evidence of the role of extracellular miRNAs as intercellular mediators, with an emphasis on their role in the mechanisms of tumor development and progression and their potential value as biomarkers in solid tumors. It also highlights the biological characteristics of extracellular miRNAs that enable them to function as regulators of gene expression, such as biogenesis, gene silencing mechanisms, subcellular compartmentalization, and the functions and mechanisms of release.

## 1. Introduction

The discovery of extracellular microRNAs (miRNAs) in blood circulation in 2008 [1,2] and, later, in other body fluids [3,4,5] in the form of highly stable, quantifiable molecules has led to growing interest and extensive research in exploring the potential use of these miRNAs as noninvasive biomarkers. Not only do extracellular miRNAs exhibit distinctive expression patterns according to body fluid in healthy individuals [4,6], but alterations in their expression profiles have been associated with several pathological conditions, including cancer [3,7,8,9,10].

Moreover, extracellular miRNAs are released by normal and cancerous cells and can be delivered to recipient cells, where they mediate the regulation of gene transcription both locally and distally [11,12,13,14]. miRNAs can be exported and imported to cells by vesicle trafficking and protein carrier mechanisms, and their role as mediators of cell-to-cell communication under normal and pathological conditions is supported by several studies.

In cancer, evidence indicates that tumor cells release miRNAs into their surroundings, especially those which are exported within vesicles such as exosomes, affecting other normal or tumor cells and promoting tumor initiation and progression. These extracellular miRNAs have been linked to the regulation of cell proliferation and migration, metastasis, epithelial–mesenchymal transition (EMT), angiogenesis, and the evasion of immune response [15]. The study of the potential role of extracellular miRNAs in oncogenesis and tumor progression not only contributes to a better understanding of the complex regulatory mechanisms of miRNA-mediated gene regulation in cancer but also provides a supportive approach for finding suitable miRNAs with diagnostic potential as noninvasive biomarkers. This review summarizes the evidence of the role of extracellular miRNAs as intercellular mediators in mechanisms of tumor initiation and progression and provides an overview of miRNAs released into peripheral blood and other body fluids as potential biomarkers in cancer, focusing on solid tumors such as lung cancer and breast cancer.

## 2. MicroRNAs

MicroRNAs (miRNAs) are small noncoding RNAs of ~22 nt that regulate the gene expression of virtually all biological processes studied to date by blocking the translation of target messenger RNAs (mRNAs). miRNAs are expressed by cells under normal physiological conditions in either a cell- or tissue-specific manner or ubiquitously, depending on the transcriptional and post-transcriptional regulation mechanisms of miRNA precursors [16,17].

### 2.1. Biogenesis

**Figure 1:**
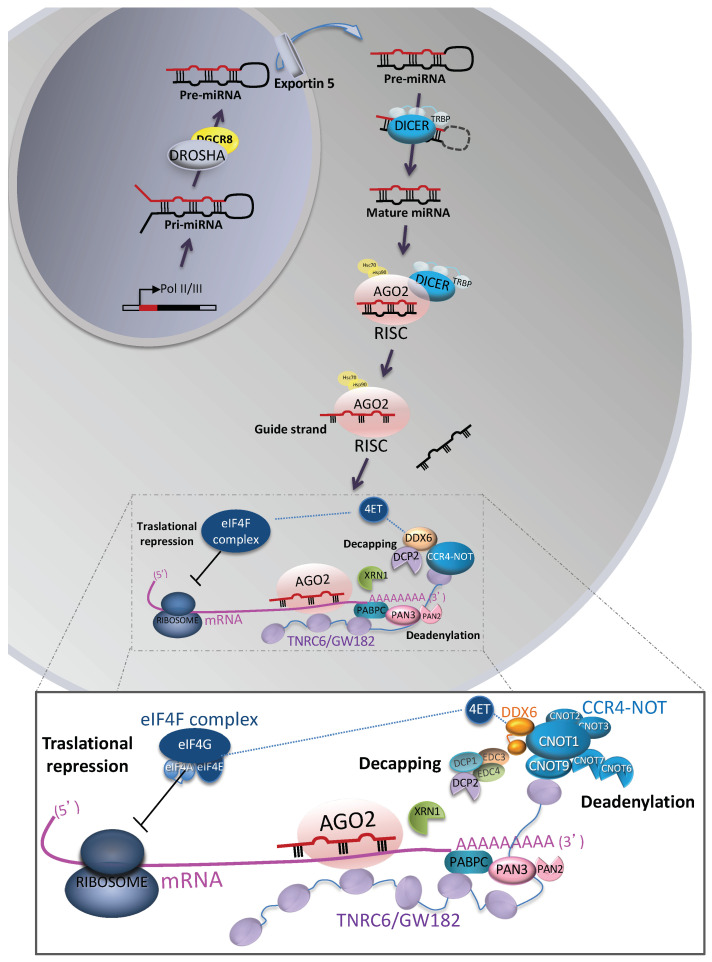
Canonical biogenesis and post-transcriptional gene silencing of microRNAs (miRNAs). In the nucleus, the miRNA gene is transcribed by RNA polymerase as a primary transcript (pri-miRNA), which is further processed by the Drosha complex to produce the precursor miRNA (pre-miRNA). Pre-miRNA is exported to the cytoplasm by exportin-5, where it is cleaved by the Dicer/TRBP complex to produce the double-stranded mature miRNA. Mature miRNA is loaded into argonaute 2 (AGO2) with the assistance of Dicer/TRBP and other proteins that form the RNA-induced silencing complex (RISC). On RISC, one miRNA strand is retained (guide strand) and the other is expulsed. miRNA binds to its target mRNA by base pairing to a partially complementary binding domain and induces gene silencing by mRNA decay and translational repression. mRNA decay involves the deadenylation and decapping of the target mRNA, followed by degradation by exoribonuclease XRN1. Deadenylation is catalyzed by PABPC and the deadenylase complexes PAN2–PAN3 and CCR4–NOT, whereas decapping is carried out by the decapping protein 2 (DCP2) with the assistance of factors DCP1, EDC3, EDC4, and DDX6. Translational repression occurs when the DDX6 interacts with 4E-transporter (4E-T), which is required for the assembly of the eukaryotic initiation factor 4F (eIF4F) complex. TRBP: transactivation response RNA binding protein; PABPC: cytoplasmic poly(A)-binding protein; EDC3 and EDC4: enhancer of decapping 3 and 4; DDX6: DEAD box protein 6.

In animals, canonical biogenesis is the dominant pathway by which miRNAs are produced (Figure 1). In this pathway, miRNA genes are transcribed as long primary precursors (pri-miRNAs) by RNA polymerase II (Pol II) [18,19]. These pri-miRNAs are cleaved by the microprocessor complex, which consists of one ribonuclease called Drosha and two molecules of the RNA-binding protein DiGeorge syndrome critical region 8 (DGCR8), to release precursor pre-miRNAs of ~70 nt [20,21]. Pre-miRNAs are exported to the cytoplasm through the action of exportin-5/RanGTP [22,23]. In the cytoplasm, pre-miRNAs are further cleaved by an enzymatic complex, formed by the endonuclease Dicer and one of the double-stranded RNA-binding protein partners transactivation response RNA binding protein (TRBP, also called TARBP2) or protein activator of the interferon-induced protein kinase (PACT), to generate mature miRNA duplexes ~22 nt in length [24,25]. Dicer/TRBP assist the loading of miRNA duplexes onto one of the four Argonaute proteins (AGO2) that are part of the protein complex, called the RNA-induced silence complex (RISC). This loading process requires also the assistance of HSC70/HSP90 chaperone proteins and the expenditure of ATP [26,27]. The complete loading RISC-complex comprises Dicer, TRBP, AGO2, and the mature miRNA duplex. Once loaded into RISC, one of the strands of the duplex miRNAs (guide strand) is retained, while the other (passenger strand) is expulsed [26,28]. Then, Dicer/TRBP may subsequently dissociate from RISC. The miRNA guide strand is now able to bind to a partial complementary sequence (imperfect pairing) located in the 3′ untranslated region (UTR) of its target mRNA [29]. However, this binding requires a perfect pairing or complementarity to nucleotides at positions 2–7 located at the 5′ end of the miRNA (“seed region”), which is essential and sufficient for miRNA function [29,30]. Structural studies revealed that the alpha-helix 7 of AGO2 leaves only the segment of miRNA corresponding to nucleotides 2–5 available for recognition [30,31,32], and when the pairing propagates to miRNA nucleotides 6–8 the alpha-helix 7 is displaced and acquires a conformation that enforces perfect pairing to nucleotides 6 and 7 [33]. Subsequently, miRNA–mRNA binding induces the repression of target transcripts through mRNA decay (mRNA destabilization) and translational repression.

### 2.2. Post-Transcriptional miRNA-Mediated Gene Silencing

The mechanism of mRNA decay involves the shortening and removal of the 3′ poly(A) tail of mature mRNAs as the initial step (Figure 1). It requires the mammalian adaptor protein trinucleotide repeat-containing protein 6B or TNRC6B (also known as GW182 in flies and nematodes), which is recruited by AGO in the RISC complex [34,35]. Then, TNRC6B interacts with the poly(A)-binding protein (PABPC) associated with the target mRNA poly(A) tail [36,37,38], followed by recruitment of the deadenylase complexes PAN2–PAN3 and CCR4–NOT [39,40]. The CCR4-NOT complex (through their component, the negative regulator of transcription 9 or CNOT9) and PAN3 bound to the tryptophan residues of TRBP. Both deadenylase complexes shorten the target mRNA poly(A) tail by consecutive and partially redundant action [41,42]. Following deadenylation, target mRNAs are decapped by decapping protein 2 (DCP2), which requires the help of decapping factors such as DCP1, enhancer of decapping 3 (EDC3), EDC4, and DEAD box protein 6 (DDX6) [43]. In metazoans, the link that couples deadenylation with decapping requires the interaction of the CCR4–NOT complex with DDX6 [44]. Finally, the decapped mRNAs are degraded by the 5′–3′ exoribonuclease 1 (XRN1) [45,46].

In addition, miRNAs repress translation by interfering with the process of ribosome scanning by targeting the eukaryotic initiation factor 4F (eIF4F) complex (Figure 1). The eIF4F complex is formed by the cap-binding protein eIF4E, the adaptor protein eIF4G, and the DEAD box eIF4A. During translation initiation, eIF4E interacts with eIF4G, which also binds the RNA helicase eIF4A and the eukaryotic initiation factor 3 eIF3 factor associated with the 40S ribosomal subunit. These interactions unwind secondary structures within mRNA 5′ untranslated regions (UTRs), allowing the 40S preinitiation complexes to scan the 5′UTR towards the start codon for translation initiation [47]. It was found that translational repression occurs through the interaction of DDX6 (RISC complex) with 4E-transporter (4E-T), a shuttling protein that binds eIF4E and competes with eIF4G, inducing the inhibition of protein synthesis by reducing the available eIF4E levels [48,49,50]. Two studies used ribosome profiling to investigate the contribution of mRNA decay (destabilization) and translational repression to miRNA-mediated gene silencing in mammalian cells [51,52]. They reported that mRNA destabilization explains most miRNA-mediated repression (66% to 90%) independently of the miRNA identity, cell type, growth condition, or translational state tested.

### 2.3. Subcellular Compartmentalization

Post-transcriptional miRNA gene silencing via RISC occurs in the cytoplasm, and it is now considered the canonical miRNA-mediated gene regulation pathway. In the cytoplasm, miRNAs, target mRNA, and RISC components are mainly located in the processing body (P-body) where miRNA gene silencing takes place [53,54]. However, they have also been found in other subcellular compartments, including the endoplasmic reticulum [55], Golgi [56], and mitochondria [57,58], where they function as regulators of gene expression [58]. Subcellular compartmentalization makes an efficient mechanism of gene silencing possible by making all the required components available by placing them in close proximity.

Most studies have indicated that miRNAs are both located and function in the cytoplasm, but recent and accumulating evidence shows that mature miRNAs are also in present and have functions in the nucleus [59,60,61,62,63], as reviewed in [64,65]. Moreover, it was found that miRNAs can mediate post-transcriptional gene silencing, transcriptional gene silencing, and transcriptional gene activation in the cellular nucleus by binding nascent RNA transcripts and gene promoter and enhancer regions [64,65,66,67,68]. These findings highlight the complex and highly regulated network of miRNA-mediated gene regulation beyond the canonical mechanisms and emphasize the relevance of the continuing research effort in this study field.

### 2.4. Functions

Studies of knockout animal phenotypes and the in vitro manipulation of miRNA expression levels have revealed the functions of most miRNAs. Analysis of knockout phenotypes has revealed that several miRNAs are required for the proper development of multiple organs such as the heart, pancreas, central nervous system, lung, skeletal muscle, vasculature, intestine, and hematopoietic lineage cells [69,70,71,72,73,74,75]. For example, the ablation of mature miRNA synthesis in the central nervous system, pancreas, or skeletal muscles of Dicer knockout mice results in nonviable mice or mice with severe developmental defects [70,71,72]. Meanwhile, in vitro studies have revealed that miRNAs are involved in the gene regulation of specific cellular processes such as differentiation, cell cycle, proliferation, apoptosis, and angiogenesis, among others [76,77,78,79,80,81].

Under pathological conditions, alterations in the miRNA expression levels are associated with a wide range of diseases, including cancer [82,83,84]. Furthermore, the in vitro and in vivo studies of disease-associated endogenous miRNAs have demonstrated their participation in the oncogenesis and tumor progression mechanisms in several cancers [85,86,87,88].

## 3. Extracellular MicroRNAs

**Figure 2:**
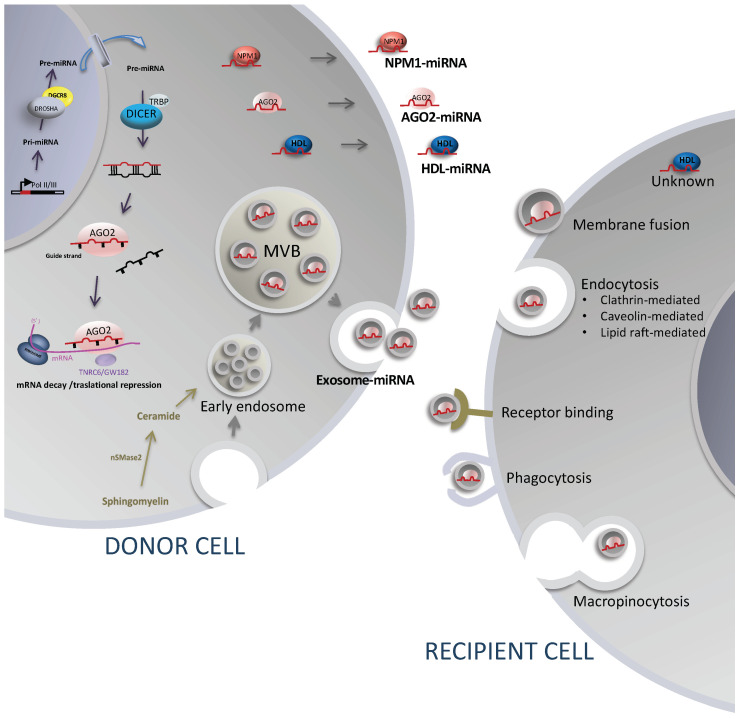
Extracellular miRNA release and uptake mechanisms between cells. miRNAs are released by cells (donor cells) through exosomes or other types of extracellular vesicles (not shown), or by complexing with argonaute 2 (AGO2), high-density lipoprotein (HDL), or nucleophosmin 1 (NPM1). Upon release into the extracellular space, exosomes can bind to the membrane of recipient cells via ligand–receptor or glycoprotein interactions, using exosome surface molecules such as tetraspanin. Recipient cells uptake exosomes carrying miRNAs by direct membrane fusion, endocytosis, micropinocytosis, phagocytosis, and receptor binding. Binding and uptake mechanisms are still unknown for HDL-miRNAs. miRNAs delivered via exosomes and HDL carrier are able to repress gene expression in recipient cells. There is no evidence of AGO2–miRNA complexes or NPM1-bound miRNAs being delivered into recipient cells.

The potential role of miRNAs as regulators in cell-to-cell communication lies in evidence indicating that endogenous miRNAs are released by cells through specific and regulated processes and delivered into recipient cells in a functional way (Figure 2). Therefore, it is relevant to review the mechanisms of release and how miRNAs are delivered into recipient cells.

Extracellular miRNAs, also called cell-free miRNAs, are released by cells via extracellular vesicles and protein/lipid carrier trafficking (a) as cargo within extracellular vesicles, especially those classified as exosomes [12]; (b) complexed with Argonaute 2 (AGO2) [89,90]; (c) bound to high-density lipoprotein (HDL) [91]; or (d) bound to the RNA-binding protein nucleophosmin (NPM1) [92] (Figure 1 and Figure 2). Since the discovery of the different carriers for extracellular miRNAs, the most extensively studied is miRNA release via exosomes.

In accordance with the notion of extracellular miRNAs as mediators of intercellular communication, it was found that miRNAs exported via exosomes [11,12,93,94] and HDL [91,95] are delivered into recipient cells in a functional way. To date, there is no evidence of AGO2–miRNA complexes or NPM1-bound miRNAs being delivered into recipient cells.

### 3.1. Release Mechanism Involving Extracellular Vesicles, Sorting, and Uptake Mechanisms

In 2018, the International Society for Extracellular Vesicles (ISEV) endorsed the use of “extracellular vesicle” (EV) as the generic term for particles naturally released from the cell that are delimited by a lipid bilayer and cannot replicate [96]. Extracellular vesicles include different particle subtypes, and each has been related to functions in cell-to-cell communication [97,98]. EVs are highly heterogeneous, making their classification very difficult [97,99]. Although there is no consensus, it has been proposed that EVs be classified based on size, subcellular origin, methods of purification, density, surface markers, and release mechanisms [96,99,100]. At least two major types of extracellular vesicles have been classified according to their subcellular origin and size: exosomes and microvesicles (also known as ectosomes or microparticles). Exosomes are small vesicles 40–150 nm in diameter that are generated by the internal budding of endosomes to produce multivesicular bodies (MVBs), which fuse with the plasma membrane, resulting in their release into the extracellular microenvironment [97,101,102]. Meanwhile, microvesicles are 150–1000 nm large extracellular vesicles generated by shedding from the plasma membrane [102,103]. Besides miRNAs, EVs contain, as cargo, biologically active RNA (including mRNA and other noncoding RNA), DNA, proteins, lipids, and metabolites [104]. They can be released into the extracellular space, where they are able to reach target cells and deliver their cargo, triggering phenotypic changes in the receptor cells [97]. “Exosomes” is the most used term in studies reporting miRNAs transported within extracellular vesicles, although the exact origin of these vesicles has not been demonstrated in many of those studies.

In 2007, Valadi et al. [12] published the first report of miRNAs within exosomes released by different cell lines that could be transferred into recipient cells. Further studies showed that miRNAs are selectively packaged into EVs, actively released, and delivered in a functional way into recipient cells [105,106,107,108].

Shortly after, it was reported that miRNAs transported within exosomes are released by tumor cells via highly regulated mechanisms and are able to affect oncogenesis and tumor progression [109,110,111,112]. The vast majority of published studies have reported extracellular miRNAs within exosomes as potential intercellular regulators of several cancer diseases.

Although the mechanisms of miRNA loading into EVs are still poorly understood, some mechanisms have been proposed based on accumulative evidence in exosomes.

Kosaka et al. [93] reported that neutral sphingomyelinase 2 (nSMase2), which triggers the formation of exosomes via the ceramide-dependent pathway, regulates the release of miRNAs transported in exosomes. In this study, the activity of nSMase2 was blocked in breast cancer cells using chemical inhibitor GW4869, which resulted in reduced miRNA release via exosomes. However, they did not provide an explanation of the sorting mechanism. On the other hand, it has been suggested that specific miRNA motifs are recognized by RNA-binding proteins as a mechanism of loading regulation. Villarroya-Beltri et al. [107] found that sumoylated heterogeneous nuclear ribonucleoprotein A2B1 (hnRNPA2B1) recognizes the EXO motif GGAG present in some miRNAs and controls their loading into the exosomes of human T cells, while Santangelo et al. [113] found that the RBP synaptotagmin-binding cytoplasmic RNA-interacting protein (SYNCRIP, also known as hnRNP-Q) recognizes miRNAs containing the hEXO motif GGCU and selectively sorts them into the exosomes of hepatocytes. Later, Hobor et al. [114] showed that SYNCRIP has a sequence-specific RNA-binding domain called NURR that mediates the recognition of the hEXO sequence in miRNA targets.

Additionally, it has been suggested that AGO2 regulates miRNA sorting. AGO2 was found to localize to multivesicular endosomes (MVEs) during exosome biogenesis, but when AGO2 is phosphorylated by the activation of KRAS–MEK signaling in colon cancer cells the AGO2–endosome association and sorting to exosomes is inhibited. The inhibition of AGO2 levels and phosphorylation affected the levels of three candidate miRNAs in exosomes (let-7a, miR-100, and miR-320a) in this study [115].

Other RNA-binding proteins have been linked to miRNA-sorting mechanisms, which include Y-box binding protein 1 (YBX-1) [116,117], MEX3C [118], major vault protein (MVP) [119], and La protein [120].

When exosomes or other EVs are released into the extracellular space, they can reach recipient cells and deliver their contents (Figure 2). How exosomes interact with the plasma membrane of recipient cells and transfer their cargoes is not fully understood. Current studies have focused mostly on exosomes, but the mechanisms are likely to be shared for different types of EVs. The data indicate that attachment to the cell surface can be mediated by exosomal tetraspanins, which can interact with integrins [121,122]. Other molecules such as heparan sulfate proteoglycans and lectins on EVs and at the plasma membrane contribute to the docking of EVs to recipient cells [122,123,124]. Once attached to the recipient cell surface, EVs can be internalized by clathrin-mediated or clathrin-independent endocytosis, such as micropinocytosis and phagocytosis, or through endocytosis via caveolae and lipid rafts [122,125,126]. On the other hand, certain types of receptors have been observed on exosomes that target specific types of cells. One example is the amyloid precursor protein on some exosomes derived from neuroblastoma cells that specifically target neurons [127]. After EVs are uptaken by recipient cells, they follow the endocytic pathway and reach the multivesicular endosomes (MVEs), which may be targeted to the lysosome. EVs that escape digestion release their cargoes into the cytoplasm of the recipient cell [128].

Interestingly, recently published papers have reported the identification of two distinct exosome subpopulations called Exo-L (large exosome vesicle of 90–120 nm) and Exo-S (small exosome vesicle of 60–80 nm), which showed distinctive biophysical and molecular properties, and the discovery of a new abundant population of non-membranous nanoparticles termed “exomeres” (~35 nm) [100,129]. These newly discovered exomeres are the predominant extracellular particles released by most cancer cells studied. These three nanoparticles subpopulations (Exo-L, Exo-S, and exomeres) were isolated from the extracellular vesicle (EV) fraction of several cancer and normal cells using asymmetric-flow field-flow fractionation (AF4), showing diverse organ distribution patterns and cargo content, which suggest specific biological functions [100,130]. Zhang et al. [100] investigated the organ biodistribution of B16-F10 melanoma-derived nanoparticles subsets in naïve mice, and found that Exo-L showed tropism for lymph nodes, whereas Exo-L, Exo-S, and exomeres were uptaken by hematopoietic organs (liver, spleen, and bone marrow), whereas lungs and kidneys showed less uptake and Exo-L, Exo-S, and exomeres were absent in the brain. Moreover, punctuated distribution patterns of nanoparticles were detected specifically in the lung and lymph nodes, in contrast to the homogenous distribution pattern found for all nanoparticle subsets in the liver, spleen, and bone marrow. Additionally, the three subsets of nanoparticles were isolated from diverse human biofluids and tissues, in which stomatin (STOM) was a specific marker detected in Exo-S and Exo-L but not in exomeres [131]. Distinct cargo types have been observed, in which Zhang et al. [100] reported that small RNA content (corresponding to tRNAs, microRNAs, and other small RNAs) was detected in Exo-S and Exo-L but not in exomeres, suggesting the absence of miRNAs in the latter. By contrast, one study [132] reported that a fraction consisting of exomeres (called distinct nanoparticles or DNPs) contained small RNAs and argonaute proteins 1, 2, and 3, suggesting the presence of miRNAs, although this was not verified. However, the authors used a modified ultracentrifugation method (instead of AF4) to isolate two fractions; one contained exosome-like particles of 94–173 nm and the second contained (DNPs) exomeres-like particles of 39–71 nm. It is possible that the fraction with DNPs may have included the Exo-S subpopulation (based in their size), but the authors did not clarify the observed discrepancy in exosome and exomere subpopulations. Nevertheless, it will be relevant to investigate whether miRNAs are present and have a role in these distinct nanoparticle subpopulations.

### 3.2. Release Mechanisms Involving Protein and Lipid Carriers

In 2011, two studies reported that a fraction of extracellular miRNAs found in human plasma and serum were exosome-free and associated with AGO2 protein [89,90]. Both studies showed that AGO2 association protects miRNAs from RNase activity, suggesting a role in extracellular miRNA stability. There is still no direct evidence that miRNAs–AGO2 complexes are uptaken by recipient cells in a functional way. In 2016, Prud’homme et al. [133] reported that neuropilin-1 (NRP1), a receptor expressed by endothelial cells, binds synthetic miRNAs, AGO2, and AGO2–miRNA complexes. They showed that NRP1 binds synthetic miRNAs with a high affinity and promotes their entry into cells. However, evidence of AGO2–miRNA uptake by recipient cells was not provided. Additionally, Fuji et al. [134] reported that human colon cancer cells released AGO2–miR-21 in the culture medium with an exponential increase in their concentration during 96 h of culture, suggesting that AGO2–miR-21 may be actively exported into the medium, but no further data were provided. The authors also reported that the AGO2–miR-21 found in the plasma of colorectal cancer patients can be used to distinguish patients from subjects without the disease.

On the other hand, in 2011 Vickers et al. [91] found that HDL transports miRNAs and delivers them to recipient cells with the functional capability of targeting messenger RNA reporters. They also found that inhibition of nSMase2 increased the cellular export of miR-223 to HDL, suggesting its role as a negative regulator. Later, in 2014 Tabet et al. [95] showed that HDL suppresses the expression of intercellular adhesion molecule 1 (ICAM-1) through the transfer of miR-223 to endothelial cells. Distinct HDL-associated miRNA profiles have been reported in the plasma of patients with familial hypercholesterolemia, atherosclerosis, stable coronary artery, acute coronary syndrome, and healthy subjects [91,135,136,137], but there are no published data regarding cancer.

In 2010, one study reported that the RNA-binding protein NPM1 binds miRNAs in the exosome-free fraction from human fibroblast HepG2 cell culture supernatants upon serum deprivation [92]. They also showed that NPM1 protects miRNAs from RNase activity. No further data have been published, nor has the detection of NPM1–miRNAs in clinical samples been reported.

## 4. Extracellular miRNAs as Cell-To-Cell Mediators in Cancer

The specific mechanisms of miRNA release into extracellular space and their presence in body fluids with a high stability led to the deduction that they may function as regulators of intercellular communication. Since their discovery, many studies have supported their role in cell-to-cell communication; however, it this remains an ongoing study area of high interest. As was mentioned earlier, many published studies have revealed a function for extracellular miRNAs transported within exosomes, while there is still little knowledge regarding EV-free miRNAs. Although extracellular miRNAs have been related to many physiological and pathological processes, one relevant issue is their role in tumor development and progression.

**Figure 3:**
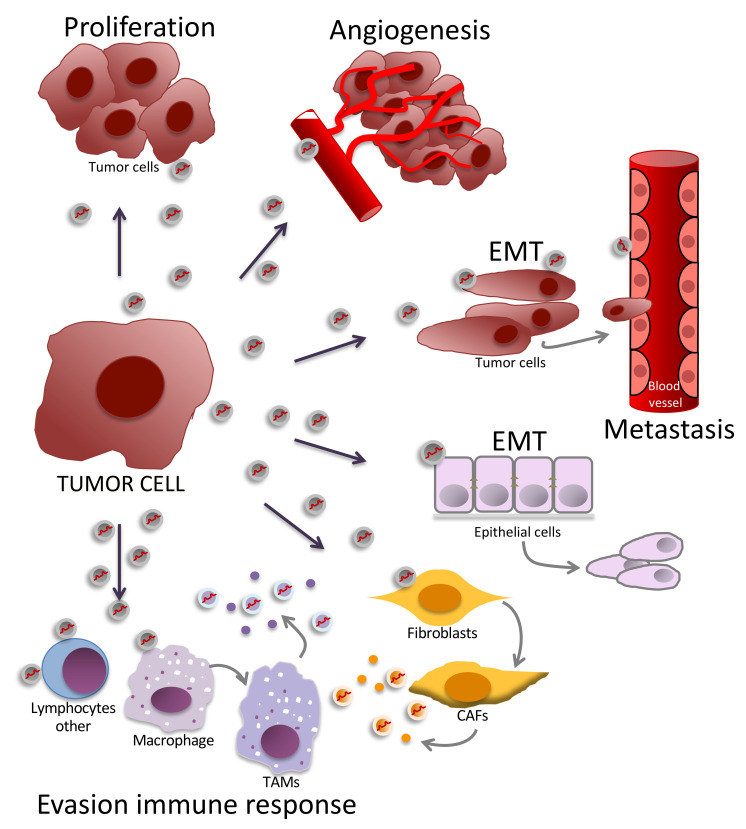
Extracellular miRNAs as intercellular mediators in cancer. In the tumor microenvironment, tumor cells release miRNAs into the extracellular space within exosomes which are delivered into other tumor cells or normal cells (resident and infiltrated cells), regulating biological processes related to cancer development and progression such as cellular proliferation, epithelial–mesenchymal transition (EMT), angiogenesis, metastasis, and the evasion of immune response. In addition to tumor cells, other cells, such as cancer-associated fibroblasts (CAFs) and tumor-associated macrophages (TAMs), release miRNAs as a mechanism of cancer regulation in addition to other soluble regulators such as cytokines as a mechanism of cancer regulation.

Briefly, tumor development involves the malignant transformation of a cell followed by establishment and invasion into the stroma of its primary tumor site (proliferation, survival). To ensure continued growth, tumor cells require nutrition and oxygen through vascular supply (angiogenesis), must evade the immune response (evasion of immune response), and finally migrate in close proximity to vascular vessels (invasion capacity, epithelial–mesenchymal transition or EMT). After vascular invasion and extravasation, metastasis occurs (invasion capacity, angiogenesis, EMT, evasion of immune response) when tumor cells reach distant sites and establish a new niche or metastatic focus in other organs and tissues [138,139,140]. Tumor development and progression involve the regulation of the cellular and tissue components of the tumor microenvironment through direct cell-to-cell contact and cell-to-cell communication via the release of biologically active molecules such as miRNAs [98,138,140]. In solid tumors, the tumor microenvironment consists not only of a heterogenous population of cancer cells but also of several other resident and infiltrated cells, diverse released factors, and components of the extracellular matrix and vasculature. Cellular components include fibroblasts, cancer-associated fibroblasts (CAFs), endothelial cells, adipocytes, immune cells, and tumor-associated macrophages (TAMs), among others [141,142]. Besides cancer cells, other abundant populations in the tumor microenvironment, such as CAFs and TAMs, participate as pro- or antitumor regulators [143,144] depending on the predominant mechanism(s) used to interact with their surroundings and the influence of the tumor microenvironment as a network [110,142,145]. Specifically, miRNA-mediated gene regulation is a relevant mechanism in the regulation of the tumor environment signaling network [110,146,147]. Evidence from in vitro and in vivo studies indicates that tumor cells deliver specific miRNAs into the extracellular space within exosomes, targeting normal and other tumor cells in the nearby microenvironment or, potentially, in remote sites (via bloodstream or other body fluids), where they regulate biological processes associated with tumor development and progression [3,146,148] (Figure 3). Moreover, evidence has revealed that other cellular components of the tumor microenvironment, such as CAFs and TAMs, export specific miRNAs that affect cancer progression-related mechanisms [147,148,149,150].

Importantly, the biological effect of either endogenous or extracellular miRNAs depends on their target gene and the target cell lineage. Accordingly, miRNAs function as pro- or anti-oncogenic gene regulators of cancer-related biological processes such as cellular proliferation, invasion capacity, metastasis, epithelial–mesenchymal transition (EMT), angiogenesis, and evasion of immune response [3,151,152] (Figure 3). An example of a pro-oncogenic miRNA (also called “oncomir” or “oncogene”) is miR-10b, which is highly expressed and also released via exosomes by the metastatic breast cancer cell line MDA-MB-231. miR-10b-enriched exosomes suppress *HOXD10* and *KLF4* gene expression in the nonmetastatic breast cancer cell line HMLE and induce HMLE cells to acquire invasive capacity [153]. An example of an anti-oncogenic (“tumor suppressor”) extracellular miRNA is miR-1. In an in vitro model of glioblastoma, miR-1 loaded into glioblastoma-derived extracellular vesicles diminished the invasion capacity and neurosphere growth of recipient glioblastoma cells in addition to the tube formation of the recipient brain microvascular endothelial cells [154]. An example of an endogenous miRNA that can function as both a pro- and anti-oncogenic regulator, depending on the cellular and target gene context, is miR-125. miR-125 can function as an oncogene in cells from hematologic malignancies [155,156] and as a tumor suppressor in cells from solid tumors [157,158]. Therefore, miRNAs can function as either pro- and anti-oncogenic mediators as either endogenous or released factors.

The next section describes recent in vitro and in vivo studies that have provided evidence of the role of miRNAs in the mechanisms of tumor development and progression, focusing on the extracellular form of miRNAs in solid tumors (Table 1).

### 4.1. Extracellular miRNAs and Epithelial–Mesenchymal Transition (EMT)

In tumors originating from epithelial cells, the epithelial–mesenchymal transition (EMT) is considered a key early step in tumor metastasis. EMT is a highly conserved and reversible process that allows epithelial cells to transition from immotile epithelial-type cells to motile mesenchymal-type cells [180,181]. In normal conditions, epithelial cells display apical–basal polarity and are bound to each other and to the extracellular matrix by tight junctions. During EMT, cells become motile and acquire invasive capacities, leading to the detachment of cells from the basement membrane. In addition, there is a loss in the expression of surface molecules such as cadherin (E-cadherin) and the expression of markers of the mesenchymal state, such as neural cadherin (N-cadherin), vimentin, and fibronectin. EMT occurs during normal development (embryogenesis) and wound healing in adults, but also during cancer progression [181,182,183]. Concerning cancer scenarios, several studies have suggested that EMT is a crucial step in the metastasis cascade that enables cancer cells to disseminate to distant sites in different carcinomas [159,184,185,186].

Regarding miRNAs, several endogenous miRNAs have been identified as regulators of EMT in cancer [160,161,162]; however, relatively limited information regarding the function of extracellular miRNAs in EMT is available.

In liver cancer, Fang et al. [148] reported that the miR-1247-3p present in exosomes derived from high-metastatic hepatocellular carcinoma cells (HCC) induced the transformation of fibroblasts to CAFs, which, in turn, promoted stemness, EMT, spheroid formation, motility, and chemoresistance in recipient HCCs in vitro, increasing in vivo lung metastasis. They found that high metastatic HCC-derived exosomal miR-1247-3p induced the transformation of fibroblasts into CAFs by downregulating B4GALT3 and through the activation of β1-integrin-NF-κB signaling in fibroblasts. When the HCC cell line SMMC-7721 was treated with conditioned medium (CM) from CAFs pretreated with high metastatic HCC-derived exosomes, they showed increases in spheroid-formation ability, motility, the expression of stemness-associated/EMT-associated genes, and resistance to sorafenib—phenomena that were partially reversed by blocking IL-6 or IL-8 using neutralizing antibodies. In vivo xenograft assays of SMMC-7721 cells treated with CM from CAFs pretreated with tumor-derived exosomes showed increased tumor growth in the lung metastases of nude mice, supporting the role of tumor-derived exosomes in metastasis. Moreover, these authors observed that high serum exosomal miR-1247-3p levels were correlated with lung metastasis in liver cancer patients.

Similarly, in pancreatic cancer Wang et al. [187] showed that the miR-301a-3p present in exosomes derived from hypoxic pancreatic cancer cells induced the M2 polarization of macrophages, which, in turn, promoted the migration, invasion, and EMT of recipient pancreatic cancer cells. Hypoxic tumor-derived exosomes containing miR-301a-3p induced M2 polarization via the activation of the PTEN/PI3kγ signaling pathway. The in vitro co-culturing of pancreatic cancer cells with M2 macrophages, in which miR-301a-3p was upregulated or treated with hypoxic tumor-derived exosomes, enhanced the migration and invasion capacity of cancer cells, induced the increased expression of mesenchymal cell markers (N-cadherin, vimentin, and MMP7), and decreased the expression of epithelial marker E-cadherin. The authors also found that exosomal miR-301a-3p facilitates the lung metastasis of pancreatic cancer cells via in vivo M2 macrophage induction. Additionally, clinical data indicate that the exosomal miR-301-3p levels in serum correlate positively with lymph node metastasis, late TNM stage, and the poor prognosis of pancreatic cancer patients.

On the other hand, miRNAs can have an opposite effect as tumor suppressors. For example, in prostate cancer Wang et al. [164] reported that the miR-26a present in exosomes derived from the low-grade prostate carcinoma cell line LNCaP inhibited cell proliferation, migration, invasion, and EMT of the metastatic castration-resistant prostate carcinoma (mCRPC) cell line PC-3. Through in vivo experiments, they also found that the tumor growth of xenograft PC-3 tumor was significantly inhibited by LNCAP exosomes. Conversely, PC-3 exosomes promoted the tumor growth of LNCAP xenograft tumor, and this promotion effect was reversed by the inhibitors of miR-26.

Meanwhile, Ota et al. [188] reported that the miR-30e transported in EVs from early-stage cholangiocarcinoma (CCA) cells suppressed EMT, cell invasion, and migration in recipient CCA cells through the targeting of Snail molecules using an in vitro model.

Other cellular components of the tumor microenvironment can contribute to tumor progression through miRNA release. For example, in lung cancer bone-marrow-derived mesenchymal stem cells (BMSCs) are a component of the lung cancer microenvironment and may contribute to cancer progression. Zhang et al. [112], using in vitro and in vivo models, demonstrated that the miR-193a-3p, miR-210-3p, and miR-5100 within exosomes derived from hypoxic BMSCs were transferred to lung cancer cells and activated the expression of mesenchymal-related molecules via the activation of STAT3 signaling-induced-EMT. The effects on metastasis were also evaluated in vivo by subcutaneously injecting C57BL/6 mice with human lung cancer cells and verifying their metastasis in lungs, which could be enhanced by injection with exosomes derived from hypoxic BMSCs. This effect was attributed to three miRNAs transported within exosomes by using specific inhibitors for those miRNAs. Another abundant cell component of the tumor microenvironment is CAF. In the context of breast cancer, Wang et al. [165] showed that CAF-derived exosomes can transfer miR-181d-5p to MCF-7 breast cancer cells and increased the proliferation, invasion, and expression of EMT markers. Using chromatin immunoprecipitation (ChIP) and dual luciferase reporter assays, they found that miR-181d-5p targets CDX2, a transcription factor binding to HOXA5 promoter. In addition, CAF-derived exosomes containing miR-181d-5p promoted the tumor growth of nude mice bearing xenografted MCF-7 cells. Similarly, but in colorectal cancer, Hu et al. [163] reported that CAF-derived exosomes containing miR-92a-3p were transferred to colorectal cancer cells (CRC), promoting their stemness, EMT, metastasis, and 5-FU/L-OHP resistance. Dual luciferase reporter assays showed that FBXW7 and MOAP1 are targets for miR-92a-3p which regulate mitochondrial apoptosis in cancer cells. In addition, the exosomal miR-92a-3p levels in serum were associated with metastasis and chemotherapy resistance in CRC patients. In prostate cancer, Josson et al. [189] showed that stromal fibroblasts overexpressing miR-409 export EVs containing miR-409 that are taken up by recipient prostate cancer cells, resulting in induced cell proliferation and EMT processes through the regulation of RSU1 and STAG2 in vitro. From in vivo experiments, they found that athymic mice subcutaneously implanted with normal stromal fibroblast overexpressing miR-409 as well as prostate cancer cells developed increased tumor volume.

### 4.2. Extracellular miRNAs and Angiogenesis

Angiogenesis is a self-limiting process that involves the formation of new capillaries from pre-existing vessels through the sprouting, proliferation, migration, and assembly of endothelial cells [167]. This process occurs during embryogenesis but mainly during postnatal life in wound healing and chronic inflammation, and it can be induced by ischemic and hypoxic conditions. In the tumor microenvironment, highly proliferative cancer cells outgrow their normal nutrient and blood supply, induce hypoxia, and produce multiple signals that promote sustained aberrant angiogenesis [3].

Several studies on exosomal miRNAs that promote tumor angiogenesis have been recently published. For example, regarding brain tumors, in 2020 Li et al. [190] reported that exosomal miR-182-5p derived from glioblastoma cancer cells under hypoxic conditions promotes angiogenesis and inhibits tight-junction-related proteins in recipient vascular endothelial cells. The authors showed that the exosomes derived from the hypoxic glioma cell lines U-251MG and U-87MG facilitate the migration and tube formation of human umbilical vascular endothelial cells (HUVEC) and enhance the endothelial permeability and transendothelial migration of tumor cells. The level of mature miR-182-5p, but not the level of pri-/pre-miR-182-5p, increased in HUVEC after they were co-cultured with exosomes derived from hypoxic U-251MG and U-87MG cells, indicating that exosomal miR-182-5p can be transferred to recipient endothelial cells. They also found that miR-182-5p targeted Krüppel-like factor 2 and 4 (KLF2 and KLF4), which induced increased promoter activity of VEGFR2 and decreased the expression of tight-junction-relevant proteins, such as claudin-5, occludin, and ZO-1. In addition, clinical data indicate that the miR-182-5p levels in exosomes from serum in patients with high-grade glioma were higher than those from patients with low-grade glioma.

Meanwhile, also in brain tumors in 2019 Wang et al. reported [191] that miR-26a overexpressed in exosomes derived from transfected glioma stem cells (GSCs) participated in the enhanced proliferation and angiogenesis of human brain microvascular endothelial cells (HBMECs) in vitro through the inhibition of PTEN. HBMECs co-cultured with exosomes containing overexpressed miR-26a had enhanced proliferation, migration, and tube formation capacity, with increased VEGF and decreased PTEN levels. The authors concluded that miR-26a targeted PTEN followed by the activation of PI3K/Akt signaling using a luciferase reporter gene assay, transfection assays of miR-26a mimic and antagonist, and analysis of the phosphorylation of PI3K/Akt in HBMEC and GSCs. Further, in vivo experiments showed that the tumor size and weight were increased in nude mice injected with GSCs transfected with miR-26a agomir and decreased with miR-26a antagomir, while the angiogenesis endothelial marker CD31 was elevated in the case of miR-26a overexpression, suggesting a role of this miRNA in angiogenesis and tumor growth promotion.

In gastric cancer, Deng et al. [166] reported in 2020 that the miR-155 present in exosomes from gastric carcinoma cells promoted angiogenesis by targeting the c-MYB/VEGF axis of endothelial cells using in vitro and in vivo experiments. Using the co-culture method, the authors found that exosomes derived from the SGC-7901 cancer cell line transported miR-155 into HUVEC, where they inhibited the expression of c-MYB but promoted the expression of VEGF, a downstream target of c-MYB. These effects almost disappeared when miR-155 was removed from SGC-7901-derived exosomes by transfecting the SGC cells with miR-155 inhibitors. Luciferase assays were used to verify that c-MYB was a target of miR-155. The biological effect observed in these co-culturing experiments was the promotion of cell proliferation, cell migration, and ring formation in HUVEC cells. These effects were not observed in exosomes derived from SGC cells transfected with miR-155 inhibitors, demonstrating the role of exosomal miR-155 in promoting angiogenesis. In vivo experiments consisted of the injection of mice with SGC-7901 cells transfected with lentivirus particles for the overexpression (OE) or knockdown (KD) of miR-155, and the results show that the miR-155 levels in serum exosomes and tumor tissues were increased in the miR-155 OE mice and downregulated in miR-155 KD mice, and were correlated with decreased levels of c-MYC and enhanced levels of VEGF mRNAs in OE mice.

In gastric cancer, Du et al. [192] reported around the same time that another exosomal miRNA (miR-23a) was released by gastric cancer cells and promoted angiogenesis by repressing PTEN with an increased expression of VEGF and a decreased expression of TSP-1. In this study, the authors used HGC-27 gastric cancer cell line-derived exosomes and HUVEC co-culture in in vitro experiments. First, they reported that HGC-27 cell-derived exosomes exhibited the highest expression of miR-23a among other gastric cell lines, and then transfected HGC-27 cells with miR-23a mimic or inhibitor and extracted the exosomes to perform co-culturing experiments. The results demonstrate the enhanced tube formation ability of HUVEC co-cultured with exosomes derived from miR-23a mimic-transfected HGC-27 cells, while tube formation was inhibited by exosomes derived from HGC-27 transfected with miR-23a inhibitor. Using a luciferase reporter gene assay and the additional transfection of HUVEC with PTEN and exosomes derived from miR-23 mimic-transfected HGC-27 cells, it was found that PTEN was inhibited by exosomal miR-23, which, in turn, decreased the levels of TSP-1 and increased the expression of VEGF in HUVEC cells and promoted angiogenesis.

In gastric cancer, in 2019 Bai et al. [168] investigated the role of FOXO1 in angiogenesis and the role of exosome-derived gastric cancer cells, focusing their research only on miR-135b because of the predicted targeted relationship between FOXO1 and miR-135b. Using both in vitro and in vivo studies, they found that the miR-135b transported in exosomes derived from another gastric cancer cell line (SGC7-901) inhibited the FOXO1 expression in HUVEC cells and enhanced the growth of blood vessels, promoting angiogenesis.

Similarly, in gastric cancer Yang et al. [169] focused their research on the role of one molecule (c-MYB) and one miRNA (miR-130a) in angiogenesis in 2018. Using vitro experiments, they found that the miR-130a present in exosomes derived from the SGC-7901 gastric cancer cell line induced decreased c-MYB expression and increased cell migration, proliferation, and ring formation in HUVEC. Similar to other studies, in vivo experiments consisted of the injection of mice with SGC-7901 cells transfected with lentivirus containing a miR-130a mimic or inhibitor, followed by the analysis of tumor growth and the miR-130a levels in exosomes isolated from the serum and the expression of angiogenesis markers in endothelial cells from mice. Their results indicate that another exosomal miR-130a promoted angiogenesis by targeting c-MYB [166].

In colorectal cancer, in 2018 Zheng et al. [146] showed that miR-25-3p, which they discovered is a metastasis-promoting miRNA of colorectal cancer (CRC), can be transferred from CRC cells (SW480 cell line) to endothelial cells (HUVEC) via exosomes. The exosome-delivered miR-25 was shown to disrupt the integrity of junctions in epithelial cells by targeting Krüppel-like factor 4 (KLF4), which induced the decreased expression of occludin, claudin-5, and ZO1, which are molecules implicated in the maintenance of cell–cell junctions. Moreover, exosome-derived miR-25-3p targeted KLF2, an inhibitor of VEGFR2, which resulted in the decreased integrity of the endothelial barrier and promoted vascular permeability and angiogenesis (increased migration and tube formation of endothelial cells). They also showed the role of exosomal miR-25-3p in vascular permeability and angiogenesis in an in vivo model. In addition, the authors showed that the high levels of serum exosomal miR-25-3p were associated with metastasis in CRC patients and with cancer diagnosis in patients with CRC compared to healthy subjects. Further, the high levels of serum exosomal miR-25-3p decreased by 85% in CRC patients after the removal of CRC tissue, indicating that tumor tissue contributed to the elevated levels of miR-25-3p in circulation.

In hepatocarcinoma, Fang et al. [170] showed that another exosomal miRNA (mir-103) increased vascular permeability by abrogating junction integrity and promoted tumor metastasis. They first identified that higher levels of serum miR-103 were associated with the metastasis of hepatocellular carcinoma. Then, using in vitro experiments, they found that hepatoma cells (QGY-7703 and HepG2 cell lines) released a high level of miR-103 within exosomes that could be delivered into endothelial cells. Using stable transfected QGY-7703 and HepG2 cells that express miR-103 in in vitro and in vivo experiments, they showed that miR-103 decreased the endothelial junction integrity by inhibiting the expression of VE-cadherin (VE-Cad), p120-catenin (p120), and zonula occludens 1.

A few studies on exosomal miRNAs with the opposite role—i.e., as suppressors of tumor angiogenesis—were also recently published. In 2018, Lu et al. [171] reported that, in nasopharyngeal carcinoma (NPC), exosomal miR-9 inhibited angiogenesis by targeting the heparin-binding growth factor Midkine (MDK) and regulating the PDK/AKT signaling pathway. Their research focused on miR-9, which they have previously identified as a tumor suppressor in NPC. They found that the miR-9 levels were reduced in exosomes derived from NPC cell lines (5-8F and CNE1) compared to a normal NP cell line (NP69). In addition, the exosomal miR-9 levels were lower in plasma samples from NPC patients compared to those from controls, and the high levels of plasma exosomal miR-9 were positively correlated with overall survival in NPC patients. Using NPC cell lines transfected with lentivirus carrying miR-9, they found that HUVEC treated with miR-9-overexpressing exosomes showed decreased migration and tube formation, a reduced level of MDK (mRNA and protein), and the reduced activation of the downstream PDK1/AKT pathway, indicating the role of miR-9 as a suppressor of angiogenesis. In 2017, Pakravan et al. [172] reported, in breast cancer, that miR-100 transported in exosomes derived from mesenchymal stem cells (MSCs) suppressed angiogenesis through the regulation of the mTOR/HIF-1a/VEGF signaling axis in breast cancer cells. This study focused only on miR-100 due to previously published evidence indicating that miR-100 is a tumor suppressor in breast cancer and is enriched in MSC-derived exosomes. The authors reported that miR-100 was present in MSC-derived exosomes. They also found that the miR-100 transcript levels were increased in MDA-MB-231 and MCF-7 breast cancer cell lines after incubation with MSC-derived exosomes, along with a decreased mRNA level of VEGF, mTOR, and HIF-1a. The VEGF mRNA levels were partially rescued in the presence of anti-miR-100, which suggested that VEGF/mTOR/HIF-1a is targeted by exosomal miR-100, but direct evidence of such targeting was not provided. In addition, HUVECs were treated with conditioned media from MDA-MB-231 or MCF-7 previously stimulated with MSC-derived exosomes, which resulted in the decreased proliferation, migration, and tube formation of HUVEC. The authors suggested that the exosomal miR-100 derived from MSCs and transferred into breast cancer cells was the cause of these biological effects in HUVEC, but direct evidence was not provided. In 2016, in gastric cancer Zhang et al. [173] found that miR-29a and miR-29c were decreased in the serum and tumor tissue of gastric cancer patients, while the levels of VEGF were elevated. Then, they identified VEGF as a direct target of miR-29a/c by using a VEGF 3′UTR luciferase assay and the transfection of gastric cancer cells (SGC-7901) with miRNA mimics or inhibitors. Finally, human embryonic kidney epithelial cells HEK293T were transfected with mimic miR-29a/c, and their cell-derived MVs were able to transfer miR-29a/c into SGC-7901 gastric cancer cells, significantly suppressing the VEGF expression and release. The co-culture of HUVEC with SGC-7901 pretreated with MVs overexpressing miR-29a/c induced a reduction in the proliferation, migration, and tube formation of endothelial cells.

### 4.3. Extracellular miRNAs and Evasion of Immune Response

The immune system in mammals is able to distinguish malignant cancer cells from normal cells and trigger a response to eliminate them. This “cancer immune surveillance” occurs when both the innate and adaptive immune responses cooperate to eradicate developing tumors. However, cancer cells “escape” immune surveillance through several escape mechanisms, allowing the continued development of tumors [193,194]. Tumor-derived miRNAs can also regulate cancer progression by modulating the immune response. Several studies have reported the role of miRNAs as regulators of the immune response in tumor microenvironments, but less information is available regarding exosomal miRNAs exported by tumor cells as a mechanism of immune response evasion. However, there have been various studies published about this in the last five years. For example, in glioma Qian et al. [174] reported, in 2020, that hypoxic glioma-derived exosomes (U87MG and U251 glioma cell lines) transferred miR-1246 into macrophages and induced M2 macrophage polarization, which, in turn, promoted glioma proliferation, migration, and invasion both in vitro and in vivo. miR-1246 induced M2 macrophage polarization by targeting TERF2IP to activate the STAT3 signaling pathway and inhibit the NF-κB signaling pathway. The same year, in melanoma Vignard et al. [175] reported that exosome-derived melanoma cells were taken up by CD8+ T cells and induced the downregulation of the T-cell response through decreased TCR signaling, diminished TNFα, and granzyme B export. The authors found that tumor-derived exosomes were enriched in miR-3187-3p, miR-498, miR-122, miR-149, and miR-181a/b. Of these, miR-181a/b and miR-498 targeted the 3′UTR sequence of *TNF*α to downregulate its expression and release by CD8+ T cells. In addition, miR-3187-3p reduced TCR signaling by directly targeting the 3′UTR sequence of *PTPRC*, which encodes CD45 that is required for TCR activation signaling. In liver cancer, in 2019 Liu et al. [176] found that miR-23a-3p was released within exosomes by hepatocellular carcinoma (HCC) cells under endoplasmic reticulum stress conditions (treated with ER stress inducer tunicamycin), which was taken up by macrophages and induced the upregulation of the immunosuppressor molecule PD-L1 in vitro and in vivo. By transfection and co-culture experiments, it was found that miR-23a inhibited PTEN expression and induced the elevated phosphorylation of AKT and PD-L1 in macrophages. The co-culture of T cells with macrophages treated with exosomes derived from the treated ER-stressed HCC cells resulted in a decreased CD8+ T cell ratio and interleukin-2 production and increased T-cell apoptosis. In liposarcoma, in 2017 Casadei et al. [177] reported that miR-24-3p and miR-92a-3p are released within EVs by liposarcoma cells and drive liposarcoma progression through the stimulation of the release of pro-inflammatory cytokine IL-6 from tumor-associated macrophages (TAMs) via a TLR7/8-dependent mechanism. They first found that miR-24-3p and miR-92a-3p were enriched in EVs from four established human liposarcoma cell lines (Lipo224, Lipo246, Lipo863, Lipo141) compared to a preadipocytes cell line (XA15A1). Then, they showed that Lipo246-derived EVs and a synthetic mix of miR-25-3p/miR-92a-3p complexed with Dotap liposomal transfection reagent induced murine macrophages to produce IL-6. Using macrophages from *TLR7*−/− mice and modified human HEK overexpressing TLR8 receptor, they showed that the IL-6 production was dependent on the presence of TLR7/8 with the subsequent activation of the NF-κB pathway. Finally, the incubation of Lipo246 cells with the supernatant of human macrophages previously incubated with Lipo246-derived EVs or a Dotap mixture of miRNAs resulted in the increased proliferation, migration, and invasion capacity of Lipo246 cells. In addition, the authors reported that high levels of miR-25-3p and miR-92a-3p in plasma were able to distinguish liposarcoma patients from healthy subjects, although the discovery cohort consisted of only 16 liposarcoma patients and 8 healthy controls. In nasopharyngeal carcinoma, Ye et al. [178] reported in 2016 that miR-24-3p enriched in exosomes derived from nasopharyngeal carcinoma (NPC) cells inhibited T-cell function. The author showed, in a previous study, that NPC-cell-derived exosomes inhibited the proliferation of T cells and the differentiation of Th1 and Th17 cells but induced the differentiation of regulatory T-cells. In this study, they focused on miR-24-3p, which was enriched in exosomes derived from NPC cell lines, and the mechanism of T-cell functional inhibition. Using an in vitro model of a transfected TW03 NPC cell line with lentivirus containing sponge RNA targeting miR-24-3p (miR-24-3p sponge) and a control (control sponge), they showed that the control sponge TW03-derived exosomes (T-EXO) inhibited the proliferation of T cells and the differentiation of Th1 and Th17 cells and induced the differentiation of regulatory T cells (Tregs), while miR-24-3p sponge T-EXO restored the proliferation index of T cells and reduced the percentage of Th1 and Th17 cells but increased the percentage of Tregs. They found that exosomal miR-24-3p directly targeted the 3′UTR of *FGF11* and the control sponge T-EXO, but not miR-24-3p sponge T-EXO, and reduced the FGF11 expression in T cells during proliferation and differentiation, indicating that exosomal miR-24-3p inhibits T cell function by targeting *FGF11*.

In addition to tumor cells, other cellular components of the tumor microenvironment can export exosomal miRNAs that can promote tumor growth by inhibiting the antitumor immune response. For example, in 2018 Zhou et al. [179] found that tumor-associated macrophages (TAMs) transfer miR-29a-3p and miR-21-5p to CD4+ T cells via exosomes and can synergistically induce the Treg/Th17 cell imbalance in epithelial ovarian cancer (EOC) through the targeting of STAT3. In a murine model of EOC, Treg/Th17 imbalance (higher distribution ratio of Treg vs. Th17) was associated with EOC primary tissue and metastatic peritoneal tissue in patients with faster tumor growth.

These recent studies provide evidence of miRNAs being released within exosomes (or EVs) by donor cells and their effect on recipient cells via the canonical binding to their target mRNAs, which ultimately modulates the immune response to the tumor.

However, in 2012 Fabbri et al. [195] reported that tumor-derived exosomal miRNAs can also function through a noncanonical mechanism of action by binding as ligands to the Toll-like receptor 8 (TLR8), which, in turn, induced a prometastatic inflammatory response. The authors identified that exosomal miR-21, miR-29a, and miR-16 were enriched in exosomes derived from non-small cell lung cancer (NSCLC) and Lewis lung carcinoma (LLC). Using coimmunoprecipitation assays, they then showed that synthetic miR-21 and miR-29a (but not miR-16) were transported within liposomes (Dotap) bound to TLR8 (found inside endosomes) in transfected human embryonic kidney (HEK-293) cells. They used a NF-κB reporter assay in TLR8–HEK-293 cells to demonstrate that NF-κB was activated by each of the tested miRNAs, with the exception of miR-16. The latter was subsequently confirmed using TLR8-HEK-293 cells transfected with a plasmid encoding a dominant negative form of TLR8 (TLR8DN) that were treated with the synthetic miRNAs, showing that the activation of NF-κB by miR-21 and miR-29a was abolished in TLR8DN cells. In addition, Dopa-miR-21 and Dopa-miR-29a induced the production of TNF-α and IL-6 in TLR8-expressing human peripheral blood mononuclear cells (PBMCs) from two healthy donors. They used peritoneal macrophages from wild type (WT) and TLR7−/− mice to show that Dopa-21 and Dopa-29a induced TNF-α and IL-6 production in WT mice, suggesting that TLR7 is required for the production of those cytokines. To assess the effect of tumor-derived exosomes, they co-cultured LLC-derived exosomes with peritoneal macrophages from WT and TLR7−/− mice and observed an increased production of TNF-α and IL-6 in WT mice. Finally, to investigate the in vivo effects, the authors silenced both the miR-21 and miR-29a expression simultaneously in LLC cells, injected these transfected cells into B6 mice, and then counted the lung multiplicities. Mice injected with LLC cells not expressing miR-21/29a in their exosomes formed fewer lung cancer multiplicities, suggesting a role in the protumoral inflammatory process in the lung. The overall data indicated that exosomal miR-21 and miR-29a, identified in exosome-derived lung cancer cells, bind to human TLR8 (and mouse TLR7) and induce the NF-kB pathway, which, in turn, increases the secretion of TNF-α and IL-6 and potentially induces an inflammatory response by macrophages that may promote tumor progression.

This new noncanonical mechanism of action of extracellular miRNAs was also reported by Challagundla et al. [196] in 2015. Using co-culture methods, the authors found that exosomal miR-21 derived from neuroblastoma cells (NBL) was uptaken by human monocytes, as an increased level of miR-21 in monocytes treated with supernatant derived from NBL cells was observed. Then, they showed that Dopa-miR-21 bound to TLR8 in human monocytes using co-immunoprecipitation assays. Moreover, NBL-derived exosomes and Dopa-miR-21 induced the upregulation of miR-155 expression in human monocytes in a TLR8-dependent manner, leading to increased levels of miR-155 in exosomes. Exosomal miR-155 was then transferred back to neuroblastoma cells, where it silenced telomeric repeat binding factor 1 (TERF1) and resulted in increased resistance to cisplatin in chemotherapy.

### 4.4. Perspective on the Biological Relevance of Extracellular miRNAs

Different investigations have proposed that extracellular miRNAs function as intercellular communicators at a paracrine level. In the context of the tumor microenvironment, this notion has been supported by a growing amount of available data. For this section, example studies were carefully chosen from the most recent published data to represent those that highlight the potential biological relevance of extracellular miRNAs in cancer biology. The rationale was that these studies provided solid evidence that the biological phenomena were due to miRNA-related paracrine mechanisms, including being able to use the most recent technology (and knowledge) available in the field.

The analysis of these studies shows that paracrine mechanisms have mainly been investigated using in vitro experiments, which are excellent experimental models but have some limitations that should be noted. The first is that in vitro experiments allow us to observe only a narrow window of the potential biological event(s). For example, if we chose a specific type of donor and recipient cell or one specific candidate miRNA, we may miss other participant cells or the effect of other relevant miRNAs in those cells. Although dissecting a biological phenomenon is necessary, we should not forget that several cellular (and noncellular) components interact with each other in the tumor microenvironment. That leads us to the second relevant limitation, which is that in vitro findings are observed outside a living organism or tissue. To compensate for these limitations, the studies discussed in this section include in vivo experiments, and some in which the miRNAs of interest are present in the serum/plasma or tissue of cancer patients. Thus, investigations that provide the best evidence to analyze the paracrine function and biological relevance of extracellular miRNAs in cancer include both in vitro and in vivo studies.

Nevertheless, challenges exist that should be taken in account. One of these is the isolation methodology of miRNA carriers—in particular, for EVs. Although different groups are working on standardizing the methodology for the isolation and classification of EVs, technical limitations remain, and there is a lack of homogenous criteria in the published studies. Additionally, it may be considered that the full characterization of EVs is not decisive for basic research; however, this could result in overlooking relevant information regarding the specific delivery and uptake mechanisms of EV-miRNAs, which would be relevant for application in translational medicine as potential therapy tools for targeted delivery to specific organs. For instance, two exosome subpopulations with distinctive biophysical and molecular properties, Exo-L and Exo-S, were recently isolated from the EV fractions of several cancer and normal cells and body fluids [100,131] (see Section 3.1). Importantly, these showed specific organ biodistribution patterns and cargo content, which suggest specific biological functions. Although these early studies suggest that miRNAs are present in Exo-L and Exo-S, further analysis has not yet been reported.

In addition, the use of artificial systems for the delivery of miRNAs in in vitro and in vivo experiments should be considered when interpreting the experimental findings because the concentration of these artificially released miRNAs may not reflect the physiological levels of extracellular miRNAs found in the microenvironment or animal system.

On the other hand, what about the biological function of extracellular miRNAs released into bloodstream and other body fluids?

An additional question can be posed about the biological function of extracellular miRNAs released into the bloodstream and other body fluids. Many studies have suggested that miRNAs in circulation function as hormone-like regulators, targeting cells in distant organs. However, some authors have argued that the levels of extracellular miRNAs are too low in body fluids to be able to induce a physiological effect on distant organs [197]. In addition, the specific uptake of exosomal miRNAs (or other carriers) by cells in distant organs may be not physiologically feasible. Moreover, the cellular origin of circulating miRNAs can be attributed to platelets or other abundant hematopoietic cells in blood [198]. Additionally, it has been suggested that the profile of miRNAs in circulation in cancer patients might be a result of the physiological response to the disease instead of the contribution of tumor cells.

The cancer-related studies described in this section did not investigate nor provide evidence of whether the exosomal-miRNAs of interest were released into the bloodstream and could affect target cells in distant organs. However, in the context of metabolism, Thomou et al. [199] demonstrated soundly that adipose tissue is a major source of exosomal miRNAs in blood circulation, and that these miRNAs regulate gene expression in distant tissues, such as the liver. In this study, mice lacking Dicer in adipose tissue were generated (ADicerKO) that exhibited reduced levels of circulating exosomal miRNAs, a reduction in white adipose tissue (WAT), the whitening of brown adipose tissue (BAT), insulin resistance, and increased levels of circulating FG21 and hepatic FGF21 mRNA. The transplantation of WAT and BAT into ADicerKO mice restored the levels of circulating exosomal miRNAs, showing that adipose tissue was the main source of those miRNAs. Furthermore, the improved glucose tolerance and reduced levels of hepatic FG21 mRNA and circulating FG21 suggest that adipose-derived miRNAs are associated with glucose regulation at the liver level. Moreover, in vitro and in vivo experiments showed that FGF21 is regulated by the serum exosomal miRNAs of normal but not ADicerKO mice. In addition, it was shown that exosomal miRNA levels were lower in sera from patients with congenital generalized lipodystrophy (CGL) and those with HIV-related lipodystrophy (who had decreased levels of Dicer in adipose tissue) compared with healthy controls. Finally, when mouse BAT was transduced with an adenovirus producing human-specific miRNA hsa_miR-302f, the exosomes present in the circulation of that mouse could target an hsa_miR-302f 3′-UTR reporter in the liver of the same mouse, or even a different mouse when given exosomes isolated from this donor.

However, our understanding of the biological relevance of circulating miRNAs in the pathophysiology of cancer progression requires further extensive investigation. For instance, have the predominant altered circulating miRNAs in cancer patients originated from neoplastic growth or, rather, are they correlated with a physiological response to the disease? In cancer patients, are the altered miRNAs transported via the bloodstream able to affect cancer-related mechanisms? Are tumor-derived exosomal miRNAs uptaken in the target cells of distant organs? Is this uptake specific? Do tumor-derived exosomal miRNAs show tropism to distant organs or tissue?

## 5. Extracellular miRNAs as Biomarker in Cancer

According to the Biomarkers, Endpoints, and other Tools (BEST) resource [200], a biomarker is defined as a characteristic that is measured and evaluated as an indicator of normal biological processes, pathogenic processes, or responses to an exposure or intervention, including therapeutic interventions.

Since the discovery of cell-free miRNAs in circulation, an increasing number of studies have aimed to identify those with diagnostic and prognosis value in solid tumors as an alternative noninvasive biomarker. To date, several extracellular miRNAs in serum and plasma (also called circulating miRNAs) have been reported as potential biomarkers for the diagnosis and prognosis of lung, breast, prostate, ovarian, bladder, pancreatic, gastric, liver, colorectal, mesothelioma, and oral cancers, among others [3,8]. Moreover, extracellular miRNAs in other body fluids, such as saliva, urine, bronchoalveolar lavage, pleural effusion, and cerebrospinal fluid, have been identified as potential biomarkers in several cancers [3].

What makes extracellular miRNAs so attractive for biomarker development is the combination of their high stability in biological samples; their relatively noninvasive obtention methods; the availability of sensitive, accurate, and reproducible measurement methods (such as quantitative PCR); and their role as intercellular mediators in mechanisms of tumor development and progression. Certainly, a cancer biomarker has to be associated with the presence of tumor cells or the malignant process itself; therefore, tumor-released miRNAs or cancer-related miRNAs are the best candidates for biomarkers.

Conventionally, many studies aiming to identify extracellular miRNAs as biomarkers in cancer have searched for the differential expression profiles of miRNAs in biological samples, mainly serum and plasma, from cancer patients compared with healthy subjects in a preclinical exploratory phase. Then, the systematic evaluation of potential candidates must be performed, which includes testing the ability of the candidate miRNAs to detect the presence of cancer with a high sensitivity and specificity in a larger and independent confirmatory study cohort. Finally, additional studies are required with large cohorts and standardized measurements to confirm the accuracy of candidate miRNAs as biomarkers in the general population. As previously mentioned, candidate miRNAs with a role in mechanisms related to tumor development or progression would have a higher potential for clinical use. Of the studies available in the literature, few have reported reaching the last phase of biomarker discovery, which is to test the research findings in the general population. Importantly, cancer is a complex and multifactorial disease, so it is unlikely that a single biomarker will be sufficient to provide a method of detection with the high rates of sensitivity and specificity required to be applied in clinics. Therefore, recent research has been conducted to identify the “signatures” of miRNAs, a set of two or more miRNAs that jointly improve the odds for future clinical use as biomarkers.

Next, we provide examples of recently published studies that investigated extracellular miRNAs in plasma, serum, and whole blood samples, which are the most extensively studied samples, as potential biomarkers in solid tumors. In a 2020 study of lung cancer, the most deadly cancer worldwide, Ying et al. [201] identified an miRNA signature in serum for the detection of early-stage non-small-cell lung cancer (NSCLC) and tested their diagnostic value in a total cohort of 744 NSCLC cases (stage I and II) and 944 matched controls,. Quantitative reverse transcription PCR (qPCR) was used for miRNA quantification in serum samples. The discovery cohort consisted of 180 NSCLC patients and 216 control subjects (Chinese male smokers). Then, they tested two independent verification cohorts consisting of 242 NSCLC patients and 190 controls (Chinese) and 101 NSCLC patients and 117 controls (Caucasians); both cohorts included males and females, and smokers and nonsmokers. From the 540 miRNAs tested, a panel of five miRNAs (let-7a-5p, miR-1-3p, miR-1291, miR-214-3p, and miR-375) were selected for distinguishing NSCLC based on their area under the curve (AUC), and they were then validated in three additional cohorts of Chinese and Singaporean patients and controls (Chinese: 120 patients vs. 117 controls; Chinese: 67 patients vs. 273 controls; and Chinese and Singaporean: 34 patients vs. 31 controls), which included stage II and IV cases. The results indicate that the five-miRNA-biomarker panel scores for each sample, calculated from a logistic regression model, were able to differentiate NSCLC cases from noncancer controls for all stages in all six study cohorts. The sensitivity was 81.3% (95% CI, 78.2–84.1%) for all cancer stages, 82.9% (95% CI, 79.8–85.7%) for stages I and II, and 83.0% (95% CI, 79.6–85.9%) for stage I NSCLC, when the specificity was 90.7% (95% CI, 88.3–92.8%), which showed that the panel has potential diagnostic value for distinguishing stages I and II NSCLS patients from matched controls, regardless of gender and smoking status.

Another example of a miRNA panel tested in a large cohort in lung cancer is the study published by Fehlmann et al. in 2020. This retrospective multicenter study aimed to evaluate the diagnostic value of miRNA signatures in whole blood samples for the diagnosis of lung cancer in symptomatic patients. This study included a total of 3046 samples from four different groups with (1) lung cancer (*n* = 606), (2) nontumor lung diseases (*n* = 593), (3) diseases not affecting the lungs (*n* = 883), and (4) unaffected control subjects (*n* = 964). Human miRNA microarrays were used to identify the candidate miRNAs; however, a quantitative method was not included in this study to validate the findings. The results reveal (a) a 15-miRNA signature (AUC 0.965) that distinguished patients with lung cancer from all other subjects in the study, (b) a 14-miRNA signature (AUC 0.977) that distinguished patients with lung cancer from nontumor lung disease patients, and (c) a 14-miRNA signature (AUC 0.960) that distinguished early-stage patients with lung cancer from subjects without lung cancer. Signature #1: miR-1285-3p, miR-205-5p, miR-1260a, miR-1260b miR-3152-3p miR-378b, miR-1202 miR-139-5p miR-16-2-3p miR-18a-3p miR-23b-3p miR-3907 miR-551b-3p miR-93-3p. Signature #2: miR-1285-3p miR-205-5p, miR-17-3p miR-1202, let-7g-3p miR-193a-5p miR-21-3p miR-3610 miR-4282 miR-4286 miR-452-3p miR-516a-3p miR-572 miR-625-5p. Signature #3: miR-1285-3p miR-205-5p miR-1260a miR-1260b miR-3152-3p miR-378b miR-17-3p, miR-564 miR-374b-5p.

Meanwhile, also in lung cancer Reiss et al. [202] investigated the diagnostic value of three miRNAs in the plasma of lung cancer patients in addition to their role in tumorigenesis, but tested a regular-sized cohort. This study included a total of 139 samples, 40 adenocarcinoma (AD), 38 lung squamous cell carcinoma (SCC), and 61 non-disease individuals, who were divided into a discovery cohort (38 patients and 21 controls) and a validation cohort (40 patients and 40 controls). This study used qPCR to quantify miRNAs in the validation cohort. The authors reported three signatures using three different statistical methods: by Elastic net (eight miRNAs: miR-16-5p, miR-92a, miR-451a, miR-106b-5p, miR-155-5p, miR-217, miR-1285-3p, miR-1285-5p), MARSA (five miRNAs: miR-16-5p, miR-148b-3p, miR-378e, miR-484, miR-664a-3p), and C-Statistic (three miRNAs: miR-16-5p, miR-92a, miR-451a). For 8-signature miRNAs, the specificity and sensitivity were 100% and 97%; for the 5-signature miRNAs, they were 97% and 100%; and for the 3-signature miRNAs, they were 100% and 84%. Finally, the authors performed bioinformatic analysis to identify molecular pathways regulated by the three chosen miRNAs, miR-16-5p, miR-92a, and miR-451a, finding that all three of them were associated with several pathways related to tumorigenesis, such as RAS, MAPK, and mTOR. However, they did not provide further experimental evidence of the association.

In ovarian cancer, the gynecologic malignancy with the highest mortality rate, several studies have investigated circulating miRNAs as noninvasive biomarkers for early diagnosis and prognosis [203,204,205]. For example, Yokoi et al. [206] published a study as part of a large-scale project in Japan aimed to develop serum miRNA-based screening cancer tests using the same platform technology (entitled “Development and Diagnostic Technology for Detection of miRNA in Body Fluids”). A total of 4046 serum samples were analyzed by a standardized microarray platform (3D-Gene^®^, Toray Industries Inc., Tokyo, Japan, with 2588 miRNA probes). Serum samples included 333 ovarian cancers, 66 borderline ovarian tumors, 29 benign ovarian tumors, 2759 non-cancer controls, and 859 solid tumors of other origin. Using randomly selected discovery and validation cohorts (160 ovarian cancer vs. 1379 non-cancer, and 160 ovarian cancer vs. 1380 non-cancer, respectively), they selected the 10 best candidate miRNAs based on the highest AUC values. Importantly, they validated the expression of candidate miRNAs by RT-qPCR and also assessed the reproducibility between microarray and RT-qPCR through analyses. Subsequently, they developed the first model for the discrimination of ovarian cancers vs. non-cancer, consisting of 10 miRNAs, with a high diagnostic performance of AUC = 1.00, sensitivity = 0.99, and specificity = 1.00. Significantly, this diagnostic accuracy was maintained in early-stage ovarian cancers. Furthermore, the authors constructed two additional models using a discovery set of 160 ovarian cancers, 120 other cancers, and 15 non-cancers, and a validation set that included 160 ovarian cancers, 739 other cancers, 100 non-cancers, 66 borderline ovarian tumors, 29 benign ovarian tumors, and 13 non-epithelial cancers. The second model consisted of a different set of 10 miRNAs that discriminated ovarian cancers from other cancer types, with an AUC = 0.87, sensitivity = 0.84, and specificity = 0.90. The third model consisted of a different set of nine miRNAs that presented a diagnostic accuracy of ACU = 0.86, sensitivity = 0.82, and specificity = 0.91, which could discriminate ovarian cancers from a non-cancer control but failed to distinguished patients with benign or borderline tumors from ovarian cancer patients. This is the largest study reported to date regarding the number of ovarian cancer and control samples included. In addition, it is the only study that included samples from other types of solid tumors.

Prostate cancer is the second most frequently diagnosed cancer and the fifth cause of death in men globally, and the accuracy of the available standard methods of detection is limited. Several studies have investigated the potential use of circulating miRNAs as diagnostic biomarkers [207,208,209,210]. An example of a large-scale study is that published by Urabe et al. [210], which is also part of the large-scale project undertaken in Japan, as previously mentioned. The authors performed miRNA profiling of 1591 serum samples using a standardized microarray platform (2588 miRNAs). The serum samples consisted of 809 histologically diagnosed prostate cancer patients (PCa), 241 negative prostate biopsies (NPBx), 500 patients with other cancer types, and 41 healthy controls. These groups were randomly divided into discovery, training, and validation sets. The discovery set included 41 prostate cancer, 41 NPBx, and 41 healthy male control samples. The training and validation sets included 384 prostate cancer and 100 NPBx samples each. Eighteen circulating miRNAs differentially expressed in prostate cancer were identified in the discovery set. Among these, a combination of two miRNAs (miR-17-3p and miR-1185-2-3p) was the best model for the discrimination of PCa from NPBx and healthy controls as indicated by the AUC reaching the optimal value (>0.99). The diagnostic performance of the model was confirmed in the training and validation sets, which in the latter, showed an accuracy of AUC = 0.95, sensitivity = 90%, and specificity = 90%. A second model for discrimination of PCa from other cancer types was developed, which consisted of a panel of 12 circulating miRNAs (miR-6471-5p, miR-17-3p, 1343-5p, miR-4417, miR-1185-1-3p, miR-1202, miR-422a, miR-6877-5p, miR-6076, miR-3185, miR-320b, and miR-1185-2-3p). This second model (AUC = 0.91, sensitivity = 91%, and specificity = 78%, in the validation set) was able to discriminate PCa from NPBx, colorectal adenocarcinoma, bone and soft tissue sarcoma, esophageal squamous cell carcinoma, and hepatocellular carcinoma with a specificity >80%, but could not successfully distinguish PCa from glioma, gastric adenocarcinoma, lung carcinoma, pancreatic cancer, biliary tract cancer, or bladder cancer. This study is the largest reported in prostate cancer to date and offers a significant advantage for further clinical application. However, the authors developed the discrimination models despite the lack of validation of the microarray data, for example by a quantitative method (qPCR), which may halt the usefulness of such models for future quantitative clinical use.

Extracellular miRNAs in circulation have also been found to be potential biomarkers for cancer metastasis. For example, in breast cancer, the second most common cancer worldwide, Shiino et al. [211] identified a serum miRNA-based prediction of axillary lymph node metastasis in breast cancer. The authors reported that a combination of two serum miRNAs (miR-629-3p and miR-4710) and three clinicopathologic factors (T stage, lymphovascular invasion, and ultrasound findings) showed a sensitivity of 88%, a specificity of 69%, an accuracy of 81.8%, and an AUC of 0.86 for the detection of axillary lymph node metastasis (N-positive) in patients with breast cancer. This was a retrospective study that included 921 patients with primary breast cancer, including 291 patients who were N-positive (32%) and 630 patients who were N-negative (68%). Patients were randomly divided into two independent cohorts: a training set of 460 patients and a test set of 461 patients. The identification of miRNAs in the serum samples was performed by microarray analysis; however, a quantitative method was not performed to validate the findings in this study. Besides the large-scale advantage of this study, the new contribution is a diagnostic model that combines a signature of serum miRNAs with clinicopathological factors, which could be a better strategy to achieve an effective diagnosis method for breast cancer metastasis.

Additionally, circulating miRNAs are potential biomarkers for predicting therapy response in cancer. For example, a study published by Li et al. in 2018 [212] reported not only a serum-based miRNA signature (miR-940, miR,451a, miR-16-5p, and miR-17-3p) that potentially predicted the response to trastuzumab of HER2+ metastatic breast cancer (MBC) patients, but also that these miRNAs were released from tumor and immune cells and targeted signaling molecules associated with trastuzumab resistance. The study design included a total cohort of 254 patients, with a training cohort consisting of 61 patients responding to the treatment and 42 resistant patients and an additional 55 healthy donors. The miRNAs present in candidates’ serum were identified with microarray analysis and further validated by qPCR. Based on the expression level of the four identified miRNAs, a disease progression risk score was calculated for each patient. The 4-miRNA-based signature was further tested in a validation cohort consisting of 151 patients (62 with a high score and 89 with a low score) and a second independent validation cohort of 132 patients (59 with a high score and 73 with a low score). The results indicate that the 4-miRNA signature was an independent prognosis factor for disease progression. Moreover, the results from in vitro experiments indicate that miR-940 was mainly released by tumor cells and miR-451a, miR-16-5p, and miR-17-3p were mainly released by immune cells via EVs. The four miRNAs were associated with the cell death of tumor cells treated with trastuzumab, which suggested that the upregulation of miR-940 and downregulation of miR-451a, miR-16-5p, and miR-17-3p induced the trastuzumab resistance of tumor cells.

Similar to studies that investigated the role of exosomal miRNAs in tumorigenesis, there are studies that investigated exosomal miRNAs (or within EVs) in circulation as potential biomarkers. For example, in colon cancer, the third most commonly diagnosed cancer worldwide, in 2020Min et al. [213] evaluated the diagnostic value of miRNAs contained within EVs obtained from plasma in early-stage colon cancer patients. The study design included a screening cohort of 15 early-stage (CC) patients and 10 normal controls (NC). Plasma EV miRNAs (let-7b-3p, miR-139-3p, miR-145-3p, and miR-150-3p) were identified by RNA sequencing and further validated using qPCR in an independent cohort consisting of 134 subjects (58 CC and 76 NC). The results show that a 3-miRNA signature (let-7b-3p, miR-139-3p, and miR-145-3p) achieved the best AUC (0.927) in distinguishing early-stage (CC) patients from normal controls. This study provided a miRNA signature with a potential diagnosis value, but further validation in a larger cohort will be required for future clinical application.

### 5.1. Perspective on Extracellular miRNAs as Cancer Biomarkers

Similar to the approach taken in Section 4, example investigations in this section were carefully chosen from the most recently published data to represent those that highlight the potential value of extracellular miRNAs as biomarkers in cancer. Therefore, the selected studies present various desirable features that include a signature or panel of extracellular miRNAs rather than a single miRNA as biomarker, large-scale validation cohorts, a clear description of the detection and quantification methods (including normalization), a clear description of biological sample manipulation, and clinical characteristics of the patients and control individuals. In addition, the tumor types in these studies were selected from those with the highest global mortality and frequency rates, which also indicate an urgent need for new noninvasive biomarker tools.

There is a rationale behind this selection. A thorough review of the available studies regarding circulating miRNAs as biomarkers in a specific type of cancer often reveals inconsistent and heterogeneous results [8,214,215]. However, the strengths and weaknesses of the research should be carefully analyzed to identify those studies that provide reliable data or those with better odds for future clinical application.

The desirable characteristics to be sought in these studies can be rationalized. Several aspects of experimental research influence the accuracy, consistency, and potential clinical value of cancer biomarker investigations. Additionally, specific technical challenges are faced in the study of extracellular miRNAs in circulation as potential cancer biomarkers. Thus, to address these it is first desirable that investigations provide complete and accurate reporting. It is then possible to identify the pre-analytical and analytical variables that affect the accuracy and consistency of the results [216], the size of study cohorts, and the use of miRNA signatures rather than a single miRNA as biomarker.

#### 5.1.1. Accurate and Complete Reporting

Clear and accurate reporting in a study, particularly of methods and results, allows readers to critically identify the strengths and weaknesses of the research. It then becomes possible to evaluate the reliability of the reported data or elucidate what further investigation is needed for potential clinical application. Importantly, incomplete reporting obstructs the evaluation of potential sources of bias in studies of diagnostic accuracy. Several studies available in the literature lack relevant information regarding design, sample collection and processing, clinical data, and the conducting and analysis of biomarker detection and quantification [217,218,219,220]. Several working groups have proposed guidelines to overcome the lack of standardized information published in the field, such as the Standards for Reporting of Diagnostic Accuracy (STARD) for diagnostic studies [221] and the Reporting Recommendations for Tumor Marker Prognostic Studies (REMARK) for prognostic studies [222]. Additionally, in the area of artificial intelligence (AI)-based technologies applied to diagnostic studies, the STARD-AI Steering Group is preparing an AI-specific extension to the STARD statement (STARD-AI) that aims to focus on the specific reporting of AI diagnostic accuracy studies [223].

#### 5.1.2. Pre-Analytical and Analytical Variables

Specific challenges related to pre-analytical variables are among those faced in the field of miRNA biomarker discovery, and include sample source (serum or plasma), the verification of the quality of samples (presence of hemolysis), storage conditions, and the spiking for RNA extraction quality control [216]. Each of these is particularly relevant for the discovery of circulating miRNA biomarkers because of their very low levels in body fluids. Even small variations in these variables may significantly alter the analysis of circulating miRNA levels.

Regarding the presence of hemolysis in the sample, it has been shown that several miRNAs are enriched in erythrocytes. Therefore, the presence of hemolysis in the sample significantly alters measurement of the levels of many circulating miRNA [216,224]. Moreover, the degree of hemolysis resulting in significantly alterations to the analysis of circulating miRNAs is low, and it is not detectable visually; therefore, other methods for detection of hemolysis should be used. The degree of hemolysis can be determined based on optical density at 414 nm (corresponding to the absorbance peak of free hemoglobin), but the best method for detecting hemolysis is the ratio of miR-451a (enriched in erythrocytes) compared to the reference miR-23a-3p [225,226]. For quality and accuracy, it is now recommended that samples are regularly examined for the detection of low-level hemolysis because hemolysis has been detected in over 40% of analyzed clinical samples [227].

Whether the study used serum or plasma samples should be verified. The results of studies using the two sample types may not be comparable, although systematic studies comparing potential differences in miRNA spectra in plasma and serum are lacking. McDonald et al. [216] reported, in 2011, that plasma contains a greater concentration of miRNAs than serum, but the authors analyzed only three miRNAs. The concentration of these miRNAs was found to be lower in plasma after first eliminating platelets by ultracentrifugation. By comparison, Wang et al. [228] reported that serum samples contain higher miRNA concentrations than plasma, and that this difference may originate from the platelet contaminant of serum samples. However, the authors showed there were no statistically significant differences between the miRNA concentrations of serum and plasma among the detectable miRNAs when using a TaqMan miRNA panel of 396 miRNAs. The differences were detected only among selected individual miRNAs measured with qPCR. In addition, the authors did not experimentally assess whether the individual miRNAs could have originated from platelets.

To determinate the recovery rate of RNA extraction in the context of quality control, it is recommended that human samples be spiked with synthetic miRNAs from other species (that do not have a human equivalent), such as *Caenorhabditis elegans* cel-miR-39.

Analytical variables are also among the specific challenges faced in miRNA biomarker discovery. Two main variables should be considered: the type of platform used for the detection and quantification of miRNAs, and the use of adequate normalizers for qPCR analysis. Platforms available for the identification and quantification of circulating miRNA include sequencing, hybridization, and qPCR-based technologies. It should be noted that the available technologies have major differences in terms of sensitivity. More specifically, sequencing and hybridization platforms have a low sensitivity and qPCR platforms have a high sensitivity and accuracy when miRNAs from serum samples are analyzed [229]. It has been proposed that the use of different platforms in different published investigations is a major reason for the lack of homogeneous results. Therefore, the use of one standardized method is recommended as an ideal, though a more practical recommendation could be that qPCR is always used for the validation of findings. Ultimately, a potential diagnostic test for clinical application should offer the high sensitivity and quantitative accuracy of qPCR.

On the other hand, droplet digital PCR (ddPCR) is a recently introduced technology that overcomes the normalization issue and may facilitate miRNA measurement. It has been reported that the ddPCR technique has some favorable features in comparison with qPCR, such as (1) absolute quantification based on the principles of sample partitioning and Poisson statistics, thus overcoming the normalization and calibrator issues; (2) increased precision and sensitivity in detecting low target copies; and (3) relative insensitivity to potential PCR inhibitors [230,231,232]. Hence, ddPCR could be a suitable option for the development of a diagnostic test for future clinical use.

Currently, the relevance of appropriate and rigorous normalization of qPCR data is well known; however, this is particularly relevant for the quantification of miRNAs from serum and plasma. In addition to the very low concentrations of miRNAs, there is no known reference miRNA in circulation that can be universally used as a normalizer. It has been demonstrated that the previous use of small nucleolar RNAs or other recommended miRNAs as a normalizer, without the verification of their stable expression, may lead to wrong conclusions or false discovery [233,234,235,236]. Therefore, the accurate quantification of circulating miRNAs by qPCR requires the identification and validation of proper reference miRNAs for the set of samples used in the study [233,236,237].

#### 5.1.3. Signatures or Panel of miRNAs

As previously mentioned, cancer is a multifactorial disease that involves genetic and epigenetic alterations in combination with environmental risk factors. In addition, the epidemiologic and genetic variability between individuals contributes to vast heterogeneity in the human population. Therefore, it is unlikely that a single biomarker will be sufficient to provide a method of detection at the high rates of sensitivity and specificity required for clinical application. Several studies have demonstrated that the use of a combination of miRNAs shows a better performance in terms of sensitivity and specificity in diagnosis than compared to an individual miRNA [201,206,210]. Conceivably, studies that propose a combination of miRNA signatures and clinical characteristics (or other kinds of diagnostic markers), such as that published by Shiino et al. [211] for breast cancer, may have better potential for clinical application. However, this hypothetical notion should be tested.

#### 5.1.4. Size of the Cohorts

Despite the large number of studies that have reported suitable circulating miRNAs as cancer biomarkers, the proposed candidates are usually considered insufficient for clinical application because of a lack of large-scale validation. The lack of statistical power of these studies is considered a major issue in the field and is attributed to the same reasons mentioned in Section 5.1.3. There are, however, various well-designed studies that include large cohorts and have reported promising results, such as those described in Section 5.

## 6. Conclusions

The accumulating evidence indicates that extracellular miRNAs, including those released into circulation and other body fluids, may function as mediators of intercellular communication in normal physiological and disease processes. In cancer, the study of the role of extracellular miRNAs in oncogenesis and tumor progression not only contributes to a better understanding of the complex pathological mechanisms involved but may also provide support for finding suitable candidates with diagnostic or prognosis potential as noninvasive biomarkers. The potential clinical value of extracellular miRNAs as potential biomarkers in cancer may rely on both their biological characteristics and their function as regulators of tumor development and progression. Therefore, further efforts are required to elucidate their precise function as intercellular communicators, in conjunction with the development of proper tools and standardized methods for miRNA quantification in body fluids. Future advances in the field of miRNAs as intercellular communicators will thus be required to overcome technical limitations, via the development of proper and standardized methodologies to investigate the transport, delivery, and uptake of extracellular miRNAs. Overcoming these challenges will allow investigators to not only elucidate the biological relevance of extracellular miRNAs but also to examine whether miRNAs within exosomes or EVs (or other carriers) are suitable tools for use in therapy, in which they can be used in the targeting and delivery of properties for specific organs and tissues affected by primary cancer or metastasis. This will elevate the field to another level, toward translational medicine, and, in particular, to personalized medicine in cancer.

## Figures and Tables

**Table 1:** Extracellular miRNAs in the mechanisms of tumor development and progression.

miRNAs	Donor Cell	Recipient Cell	Molecular Targets	Function	Tumor Type	Ref
miR-1247-3p	High-metastatic hepatocellular carcinoma cells (HCC)	Fibroblasts	B4GALT3, β1-integrin-NF-κB signaling	Induce the transformation of fibroblasts to CAFs. CAFs promote stemness, EMT, motility, and chemoresistance in recipient HCCs.	Liver cancer	Fang et al. [144]
miR-301a-3p	Hypoxic pancreatic cancer cells (PCC)	Macrophages	PTEN/PI3kγ signaling	Induce the M2 polarization of macrophages, which promotes the migration, invasion, and EMT of recipient PCCs.	Pancreatic cancer	Wang et al. [159]
miR-26a	Low-grade prostate carcinoma cell line LNCAP	Metastatic castration-resistant prostate carcinoma cell line PC-3	ND	Inhibit the cell proliferation, migration, invasion, and EMT of PC-3.	Prostate cancer	Wang et al. [160]
miR-30e	Early-stage cholangiocarcinoma (CCA) cells	CCAs	Snail	Suppress EMT, cell invasion, and migration in recipient CCA.	Cholangiocarcinoma	Ota et al. [161]
miR-193a-3p, miR-210-3p, and miR-5100	Bone-marrow-derived mesenchymal stem cells (BMSCs)	Lung cancer cells	STAT3	Induce EMT and metastasis.	Lung cancer	Zhang et al. [112]
miR-181d-5p	CAFs	MCF-7 breast cancer cells	CDX2 and HOXA5	Increased proliferation, invasion, and expression of EMT markers.	Breast cancer	Wang et al. [162]
miR-92a-3p	CAFs	Colorectal cancer cells (CRC)	FBXW7 and MOAP1	Promote stemness, EMT, metastasis, and 5-FU/L-OHP resistance.	Colorectal cancer	Hu et al. [163]
miR-409	Fibroblast	Prostate cancer cells	RSU1 and STAG2	Induce cell proliferation and EMT.	Prostate cancer	Josson et al. [164]
miR-182-5p	Hypoxic glioblastoma cancer cells	Umbilical vascular endothelial cells (HUVEC)	KLF2 and KLF4	Promote angiogenesis and inhibit tight-junction-related proteins.	Glioblastoma cancer	Li et al. [165]
miR-155	SGC-7901 gastric cancer cells	HUVEC	c-MYB/VEGF	Promote angiogenesis.	Gastric cancer	Deng et al. [166]
miR-23a	HGC-27 gastric cancer cells	HUVEC	PTEN and TSP-1	Promote angiogenesis.	Gastric cancer	Du et al. [167]
miR-135b	SGC7-901 gastric cancer celsl	HUVEC	FOXO1	Promote angiogenesis.	Gastric cancer	Bai et al. [168]
miR-130a	SGC-7901 gastric cancer cells	HUVEC	c-MYB	Increase cell migration, proliferation, and ring formation in HUVEC.	Gastric cancer	Yang et al. [169]
miR-25-3p	SW480 CRC cells	HUVEC	KLF4 and KLF2	Disrupt the integrity of junctions in epithelial cells.	Colorectal cancer	Zheng et al. [146]
mir-103	QGY-7703 and HepG2 hepatocarcinoma cell lines	Endothelial cells	VE-Cad, p120, and zonula occludens 1.	Increase vascular permeability by abrogating junction integrity and promoting tumor metastasis.	Liver cancer	Fang et al. [170]
miR-9	Nasopharyngeal cancer cells	HUVEC	MDK and PDK/AKT signaling	Inhibit angiogenesis.	Nasopharyngeal carcinoma	Lu et al. [171]
miR-100	Mesenchymal stem cells (MSCs)	Breast cancer cells	mTOR/HIF-1a/VEGF	Decrease the proliferation, migration, and tube formation of HUVEC.	Breast cancer	Pakravan et al. [172]
miR-29a and miR-29c	SGC-7901 gastric cancer cells	Endothelial cells	VEGF	Decrease the proliferation, migration, and tube formation of endothelial cells.	Gastric cancer	Zhang et al. [173]
miR-1246	Hypoxic glioma cells	Macrophages	STAT3 and NF-κB signaling	Induce M2 macrophages, which promote the glioma proliferation, migration, and invasion of glioma cells.	Glioma	Qian et al. [174]
miR-181a/b, miR-498	Melanoma cells	CD8+ T cells	PTPRC (CD45)	Decrease TCR signaling, TNFα, and granzyme B secretion.	Melanoma	Vignard et al. [175]
miR-23a-3p	HCC cells under endoplasmic reticulum stress	Macrophages	PTEN, AKT, PD-L1	Upregulation of the immunosuppressor molecule PD-L1.	Liver cancer	Liu et al. [176]
miR-24-3p miR-92a-3p	Liposarcoma cells (LSC)	Tumor-associated macrophages (TAMs)	TLR7/8, NF-κB pathway	Secretion of pro-inflammatory cytokine IL-6 by TAMs, which promote the proliferation, migration, and invasion capacity of LSCs.	Liposarcoma	Casadei et al. [177]
miR-24-3p	NPC cells	T cells	FGF11	Inhibit the proliferation of T cells, inhibit the differentiation of Th1 and Th17 cells, and induce the differentiation of Treg cells.	Nasopharyngeal carcinoma	Ye et al. [178]
miR-29a-3p miR-21-5p	TAMs	CD4+ T cells	STAT3	Treg/Th17 imbalance.	Epithelial ovarian cancer	Zhou et al. [179]

ND: not determined.
